# Galectin-3 inhibition ameliorates hepatic steatosis in a multilineage 3D spheroid model

**DOI:** 10.1371/journal.pone.0326373

**Published:** 2025-07-03

**Authors:** Francesca Sedda, Andrea Caddeo, Kavitha Sasidharan, Giovanni Perra, Rajesh Pal, Nicola Lai, Marta Anna Kowalik, Andrea Perra

**Affiliations:** 1 Unit of Oncology and Molecular Pathology, Department of Biomedical Sciences, University of Cagliari, Cagliari, Italy; 2 Wallenberg Laboratory, Sahlgrenska Academy, Institute of Medicine, Department of Molecular and Clinical Medicine, University of Gothenburg, Gothenburg, Sweden; 3 Department of Mechanical, Chemical and Materials Engineering, University of Cagliari, Cagliari, Italy; 4 Computational Oncology Group, Molecular Precision Oncology Program, German Cancer Research Center (DKFZ) and National Center for Tumor Diseases (NCT) Heidelberg, Heidelberg, Germany; THe University of Texas in Austin, UNITED STATES OF AMERICA

## Abstract

**Background:**

Metabolic dysfunction-associated steatotic liver disease (MASLD) is the leading cause of chronic liver disease, and liver-related morbidity and mortality worldwide. MASLD is a multifactorial condition, which still needs to be completely understood. Galectin 3 (Gal-3) is up-regulated in several liver disorders suggesting its implication in the mechanisms underlying liver damage.

**Methods:**

A human multilineage 3D model was utilized to investigate the role of Gal-3 in MASLD development. Human hepatoma cell line (HepG2) and human stellate cell line (LX-2) were co-cultured in a physiological ratio of 24:1 and treated with a mixture of palmitic and oleic acid (PAOA, ratio 1:2) to induce hepatocyte steatosis and facilitate the development of fibrosis. While the effect of *LGALS3* silencing on neutral fat content was assessed by Oil-Red-O (ORO) staining, type I collagen production was analysed by immunofluorescent staining for collagen type I alpha 1 (COL1A1).

**Results:**

Gal-3 depletion caused a reduction of neutral lipid content and COL1A1 accumulation in 3D spheroids. While *LGALS3* silencing did not significantly alter the respiratory state, analysis of genes involved in lipid metabolism demonstrated significant changes in genes involved in β-oxidation and triglyceride synthesis.

**Conclusion:**

These results suggest a role of Gal-3 in the regulation of fatty acid and collagen accumulation, thereby indicating that approaches aimed at inhibiting Gal-3 may represent a promising therapeutic strategy in MASLD.

## Introduction

Galectin 3 (Gal-3), previously known as MAC2, is a chimera type galectin, that belongs to the family of β-galactoside-binding proteins, involved in mRNA splicing, cell cycle, cell growth and cell apoptosis [1–3]. Gal-3 is a 30 kDa protein highly expressed in gastrointestinal tract, skin, bone marrow and lymphoid tissue, including all types of immune cells, sensory neurons, epithelial and endothelial cells [4–6]. On a subcellular level, Gal-3 displays specific functions depending on its location, as it predominantly locates in the cytoplasm, but can shuttle into the nucleus, or even be secreted to the cell surface or into biological fluids [1]. Gal-3, by binding to the cell surface and extracellular matrix glycans, affects a variety of pathologic processes, such as lipid metabolism, inflammation, and tumour development [3,6–17].

Metabolic dysfunction-associated steatotic liver disease (MASLD), previously known as non-alcoholic fatty liver disease (NAFLD), is the leading cause of chronic liver disease (CLD) and liver-related morbidity and mortality worldwide [18]. MASLD, characterized by hepatic fat accumulation and co-existing cardiometabolic risk factors, is often clinically associated with liver fibrosis, with a massive activation of the hepatic stellate cells (HSCs) into myofibroblasts-like cells that secrete large amount of extracellular matrix (ECM) proteins [19,20]. ECM remodelling towards a fibrotic phenotype represents an important risk factor for hepatocellular carcinoma (HCC). HCC incidence and mortality are continuously increasing, and, unfortunately, the approved pharmacological therapies are still poorly effective [21]. While Gal-3 is not expressed by hepatocytes in the healthy liver [22], elevated serum levels of Gal-3 have been observed in individuals with steatotic liver disease, metabolic-associated steatohepatitis (MASH), fibrosis, cirrhosis, and HCC [23,24]. Recently, Resmetirom (Rezdiffra, Madrigal Pharmaceuticals, Inc), a thyroid hormone receptor-beta (THRβ) agonist was approved for the treatment of non-cirrhotic MASH with moderate to advanced liver fibrosis [25]. Considering the complexity of MASLD pathophysiology, a single drug might not be effective in all patients or in all stages of the disease. For this reason, combination therapies are needed. A possible approach is the development of drugs that can be associated with Resmetirom, but acting on different molecular targets [26]. In this regard, Belapectin (GR-MD-02), a Gal-3 inhibitor, is currently under phase 2b/3 clinical trial after exhibiting reduced hepatic venous pressure gradient and development of varices [27].

Initial *in vivo* studies demonstrated that the absence of the gene encoding for Gal-3, namely *Lgals3*, led to hepatic steatosis in mice [28]. *Lgals3*^−/−^ mice, at the age of 15 months, developed liver nodules with nuclear atypia associated with mild to moderate zone 3 fibrosis [29]. Another study reported that *Lgals3*^−/−^ mice fed a high fat diet (HFD) were more subjected to liver fat accumulation, without signs of hepatocellular injury and liver inflammation [30]. In spite of contrasting data, depending on the experimental design, the analysis of multiple models of fibrosis showed that Gal-3 is potently a pro-fibrotic factor able to modulate the activity of stellate cells/fibroblasts and macrophages in chronically inflamed liver [31].

Preclinical *in vitro* models, that range from monolayer (2D) cell cultures of immortalized hepatic cell lines to three-dimensional (3D) co-cultures of different cell populations in the same microenvironment [32], have significantly enhanced our understanding of mechanisms involved in liver disease development and progression. 3D *in vitro* models are emerging as a relevant tool to bridge the gap between 2D cell cultures, that have been the main *in vitro* system for studying MASLD for many years, and *in vivo* models. In the last decade, the number of studies based on 2D approaches alone decreased; in fact, the use of a single cell line lacks the complex interactions between the different cell types that make up the organ. In this specific case, the study of MASLD cannot be limited to the analysis of the mechanisms involving hepatocytes, without evaluating their interaction with non-parenchymal cells [33]. Recently, multilineage 3D spheroids, consisting of HepG2 and LX2 cell lines, homozygotes for the *PNPLA3* I148M sequence variant, the strongest genetic determinant for MASLD, have been developed [34]. This important tool allows the interaction of hepatocytes with non-parenchymal cells and can be used to study the molecular mechanisms involved in hepatic lipid accumulation, early stages of fibrogenesis, as well as to identify new compounds for the treatment of MASLD.

In this study, we employed human multilineage 3D spheroids to elucidate the role of *LGALS3* in the development of hepatocyte steatosis and fibrosis.

## Materials and methods

### Cell lines

Human hepatoma cell line (HepG2) (ATCC, Menassas, VA, USA) were plated in T-75 flasks with Minimum Essential Medium (MEM) supplemented with 10% Fetal Bovine Serum (FBS), L-glutamine 2 mM, sodium pyruvate 1 mM, non-essential amino acids 1X, penicillin 100 units/mL, and streptomycin 100 µg/mL (HyClone Laboratories, Logan, UT, USA). Immortalized human hepatic stellate cell line (LX-2) (Millipore, Burlington, MA, USA) were plated in T-75 flasks with high glucose Dulbecco’s Modified Eagle Medium (DMEM) (HyClone Laboratories) supplemented with 10% FBS, penicillin 100 units/mL, and streptomycin 100 µg/mL (Cytiva, Marlborough, MA). Cell lines were maintained at 37 °C in a humidified atmosphere of 5% CO_2_.

### Spheroid culture

Spheroids composed of HepG2/LX2 cells with a 24:1 ratio were cultured in 96-well round bottom ultra-low attachment plates (Corning, Camelback Rd. Glendale, AZ, USA) and were grown in MEM supplemented as described above. The plates were incubated for 96 h at 37 °C in a humidified atmosphere of 5% CO_2_ [34].

### Gene silencing

Directly after seeding, HepG2/LX2 cells with a 24:1 ratio were transfected with either scramble (SCR) (Thermo Fisher Scientific, Waltham, MA, USA) or a mix of three *LGALS3* small interfering RNA (siRNA) (s8148, s8149, s8150, Thermo Fisher Scientific) at a final concentration of 20 nM, through the employment of Lipofectamine^TM^ 3000 Transfection Reagent (L3000015, Thermo Fisher Scientific), following manufacturer’s instructions.

### Fatty acid treatment

48 hours after seeding, spheroids were incubated with a mix of palmitic and oleic acid (PAOA 1:2) conjugated to BSA 1%, to a final concentration of 500 µM (Sigma-Aldrich, St. Louis, MI, USA) [34]. The media of the control group was supplemented with 1% bovine serum albumin (BSA). For conjugation, fatty acids were dried at 42 °C and resuspended in medium (1/10 of the desired volume) containing 10% BSA and mixed overnight at 37°C. The day after, the medium was diluted 1:10 with fresh medium, to obtain the established concentration of fatty acids.

### Viability assay

Viability assay was tested on 2D cells by using the CellTiter 96^R^ AQueous One Solution cell proliferation Assay kit (Promega G3582, Madison, WI, USA) according to the manufacturer’s instructions. Viability of the spheroids was assessed through the measurement of ATP content as previously described [34]. CellTiter-Glo Luminescent Cell Viability Assay kit (Promega, Madison, WI, USA) was used according to the manufacturer’s instructions. SpectraMax i3 counter (Molecular Devices, Thermo Fisher Scientific) was employed and luminescence was measured by using the SoftMax Pro 6.3 software (San Jose, CA, USA).

### RNA extraction and qRT-PCR

Total RNA was extracted using RNeasy Plus Mini Kit (Qiagen, Hilden, Germany) and reverse-transcribed using a High-Capacity cDNA Reverse Transcription Kit (Thermo Fisher Scientific). Gene expression analysis was performed using TaqMan Gene expression Master Mix (Thermo Fisher Scientific) and the specific TaqMan probes: *LGALS3* Hs00173587_m1, *ACACA* Hs01046047_m1, *ACACB* Hs01565914_m1, *APOB* Hs00181142_m1, *CD36* Hs00354519_m1*, CPT1A* Hs00912671_m1, *CPT2* Hs00988962_m1*, DGAT1* Hs01020362_g1, *DGAT2* Hs01045913_m1, *ELOVL6* Hs00907564_m1, *FASN* Hs01005622_m1, *LDLR* Hs01092524_m1*, MLXIPL* Hs00975714_m1, *MTTP* Hs01055363_m1, *PPARA* Hs00947536_m1, *PPARG* Hs01115513_m1*, SCD* Hs01682761_m1, *SREBF1* Hs02561944_s1, *UCP1* Hs01084772_m1 and *ACTB* Hs01060665_g1 (Thermo Fisher Scientific) as an endogenous control. Each sample was run in triplicate, and all measurements were normalized to β-actin. Relative mRNA expression analysis was calculated by using the 2^−∆∆Ct^ method.

### Spheroids imaging

After 2 hours of fixing with 10% v/v paraformaldehyde (PFA, Sigma-Aldrich) in Phosphate Buffered Saline (PBS, Lonza, Basel, Switzerland), spheroids were incubated with 20% w/v sucrose in PBS overnight and embedded in OCT Cryomount (Histolab, Västra Frölunda, Sweden) after washing with PBS. The spheroids were sectioned into 8 µm-thick slides using a cryostat (Leica, Wetzlar, Germany). Both spheroids and sections were stored at −80 °C.

### Oil Red-O staining

Oil Red-O (ORO, Sigma Aldrich) staining was used to visualize the hepatic neutral lipid content. A rinse with 20% isopropanol was performed before and after the incubation of the cells/slides with ORO working solution for 20 minutes. Nuclei were stained with DAPI (Sigma-Aldrich) (1:8000 in PBS) for 5 minutes. Finally, cells were mounted with fluorescence mounting medium (Dako). Images were obtained using Axioplan 2 (Zeiss, Oberkochen, Germany) with AxioVision 4.8 Software (Zeiss), applying a static threshold to all images for each of the fluorescent channels to determine positively stained area. Image analysis was performed using an in-house macro in ImageJ (v.1.52h, NIH) counting nuclei and the total spheroid area.

### Immunofluorescence

Sections were incubated with primary antibody anti-COL1A1 (HPA011795, Sigma-Aldrich) (1:100) diluted in 5% w/v BSA (PBS) for 1 hour at room temperature (RT), after the blocking of unspecific sites with 5% w/v BSA (Sigma-Aldrich) in PBS for 1 hour. Subsequently, they were washed twice and incubated with a fluorescent secondary antibody (Alexa Fluor 594, 1:2000, Invitrogen by Thermo Fisher Scientific) for 1 hour at RT. Nuclei were stained with DAPI (Sigma-Aldrich) (1:8000 in PBS) for 5 minutes. Image analysis has been done as described before.

### High-resolution respirometry

Mitochondrial respiration rate was measured in cell suspension by a polarographic system (OROBOROS-O2k, Innsbruck, Austria). HepG2 cells were suspended in MiR05 (0.5 ∙ 10^6^ cells ml-1) and transferred to the chamber of the polarographic system with a total volume of 2 ml. Datlab software (OROBOROS Instruments) was used for data acquisition and to calculate O_2_ consumption rate in the cell suspension. The temperature and stirring of the metabolic chamber were maintained at 37°C and 750 rpm, respectively. Cell respiration protocols started following stabilization of the O_2_ consumption rate of the intact cell basal respiration (ICR). Respiration rate was expressed in pmol s-1 10^-6^ cells and measured in an O_2_ concentration range of 75–195 μM. The oxidative phosphorylation (OxPhos) protocol was used to evaluate mitochondrial function with complex I (C-I, NADH-dehydrogenase), II (C-II, succinate dehydrogenase), and IV (C-IV, cytochrome c oxidase) substrates, uncoupler, and inhibitors with the following [35,36]: malate (2 mM), pyruvate (2.5 mM), digitonin (2.5 μM), ADP (2.5 mM), succinate (10 mM), (CCCP; titration up to an optimum concentration 0.5–12.5 μM), rotenone (2 μM), antimycin A (0.5 μM), ascorbate (2 mM), TMPD (0.5 mM), and sodium azide (200 mM). Prior to the OxPhos protocol specific experiments were performed to determine the optimum digitonin concentration by titration [36]. The difference between the uncoupled mitochondrial respiration rate (ET) and that in the presence of rotenone (R), which quantifies mainly the contribution of C-II to uncoupled oxidation, was used to determine NADH-linked respiration rate (ET-R), while the azide sensitive respiration rate in the presence of ascorbate plus TMPD was used to determine complex IV respiration rate (C-IV). Respiration rate in the presence of antimycin A was subtracted from all mitochondrial respiration rates.

### Western blotting analysis

HepG2 and LX-2 cells were washed with PBS and lysed in ice using RIPA buffer (Sigma-Aldrich) supplemented with protease and phosphatase inhibitors (Sigma-Aldrich). Protein samples were mixed with Laemmli Buffer, boiled at 95 °C for 5 minutes, size-separated by SDS-PAGE (sodium dodecyl sulfate–polyacrylamide gel electrophoresis) gels (12% acrylamide) and then transferred onto nitrocellulose membrane by Trans-Blot Turbo Transfer Pack (Biorad). The immunoblots were incubated with 5% (w/v) non-fat dry milk in TBS-T buffer (Tris-buffered saline containing 0.2% Tween) at room temperature for 1 hour and then probed with primary (anti-Gal-3 antibody ab76245, Abcam; anti beta-Actin antibody ab8226, Abcam) and appropriate secondary (mouse anti-rabbit IgG-HRP sc-2357, Santa Cruz) antibodies. Membranes were incubated with chemiluminescent substrate (SuperSignalTM West Pico PLUS Ref 34580, Thermo Fisher) for 5 minutes, bands were visualized and quantified by iBrightTM CL750 Imaging System (Invitrogen by Thermo Fisher).

### Statistical analyses

All data were expressed as mean ± standard deviation (SD). Differences between groups were compared using unpaired two-tailed Student’s t-test ANOVA or Tukey test using the GraphPad Prism version 8.0.2 software (La Jolla, California, USA,). P-values were considered significant at P < 0.05. Respiration rates were expressed as mean ± standard deviation (SD). Statistical significance was set at a p-value <0.05 and analysed using OriginPro 2024. Two-way ANOVA with Holm-Bonferroni correction for multiple comparisons was used to investigate the respiratory state and *LGALS3* silencing effects on the respiration rate of HepG2 cells. For data presented in Supplementary Fig 1, the Wilcoxon Rank-Sum Test was used to test *LGALS3* expression levels between different groups.

## Results

### Analysis of *LGALS3* expression in hepatoma cell lines

**Fig 1:**
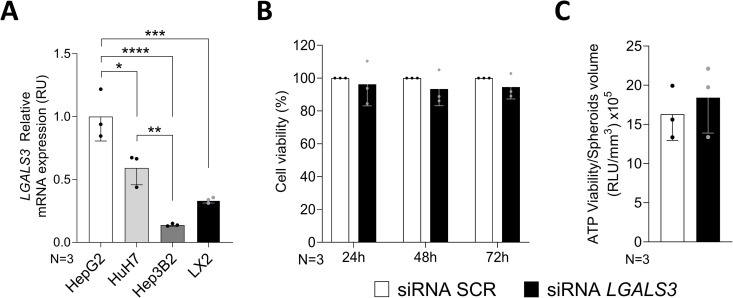
Cell viability is not affected following *LGALS3* silencing. A. *LGALS3* mRNA expression levels in human hepatoma (HepG2, Huh7, Hep3B2) and hepatic stellate (LX-2) cell lines. All measurements were normalized to β-actin. Relative quantification was calculated by 2^‐ΔΔCt^ method. Groups were compared by using one-way ANOVA followed by Tukey *post hoc* analysis. B. HepG2 cells viability was not modified in siRNA SCR and siRNA *LGALS3* after 24, 48 or 72 hours of silencing. The viability was expressed as percentage normalized to the siRNA SCR, from the absorbance measured at 490 nm. C. Spheroids viability was not affected by the *LGALS3* ablation. The viability was expressed as percentage of ATP normalized to the spheroids volume. The volume was determined by the following formula: 4/3 π r 3, where “r” was the mean of the long diameter and short diameter of the spheroid divided by two. Graphs represent N = 3 independent experiments. * p < 0.05, ** p < 0.01, *** p < 0.001, **** p < 0.0001. Bars represent mean ± SD. Abbreviations: SCR, scramble.

As previously reported [37], over 20 hepatoma cell lines expresses detectable levels of *LGALS3.* Using the Human Protein Atlas (Version 23.0) to verify *LGALS3* expression levels in hepatoma cell lines, HepG2, HuH7, and Hep3B2 cell lines were selected. *LGALS3* mRNA levels of these cell lines were validated by qRT-PCR. Additionally, the expression levels in a human hepatic stellate cell line (LX2) have been examined (Fig 1A). HepG2 cell line, which exhibited the highest levels of *LGALS3* mRNA, was selected to carry out the successive silencing experiments.

### *LGALS3* silencing does not affect cell viability

Successively, viability test was assessed in both HepG2 2D cultured and 3D spheroids following *LGALS3* knockdown. (3-(4,5-dimethylthiazol-2-yl)-5-(3-carboxymethoxyphenyl)-2-(4-sulfophenyl)-2H-tetrazolium) (MTS) assay was performed on HepG2 cells and the number of live cells was measured at three different time points: 24, 48 and 72 hours after silencing. Spheroids viability was verified at the end of the usual experimental time (96 hours) by ATP measurement normalized by the volume of the spheroids. As evident from Fig 1B–1C, cell viability was not affected by *LGALS3* silencing.

### Effect of *LGALS3* silencing on mitochondrial energetics

**Fig 2:**
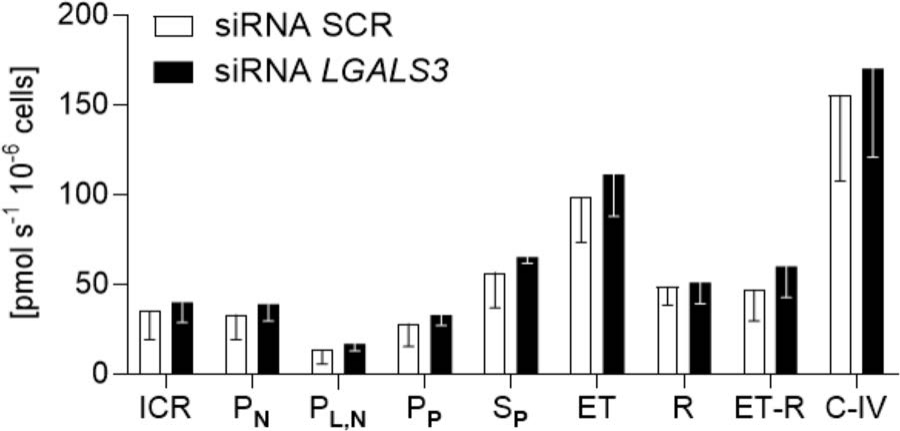
Effect of *LGALS3* silencing on mitochondrial energetics. Mitochondrial respiration rates of permeabilized siRNA SCR and siRNA *LGALS3* HepG2 cells (0.5 ∙ 10^6^ cells ml-1) obtained with intact cell (BR), malate and pyruvate (PN), permeabilized cell with digitonin (PL,N), OxPhos state with malate and pyruvate (PP), succinate (SP), uncoupled mitochondria to measure maximal electron transport chain capacity with CCCP (ET), rotenone (R), (ET-R) is contribution of the C-I on respiration of uncoupled mitochondria (ET) and also ET-R indicates the respiration rate difference between that indicated with ET and R, C-IV indicates the respiration rate difference between that with ascorbate plus TMPD and azide. Mitochondrial respiration rate data are mean ± SD.

The impact of *LGALS3* silencing on the mitochondrial bioenergetics was assessed on permeabilized HepG2 cells in the presence of ETC substrates (Fig 2**).** Permeabilized siRNA SCR and siRNA *LGALS3* HepG2 cells were characterized by measuring the activities of the main mitochondrial complexes (C-I, C-II, and C-IV) (Fig 2). No significant differences in the respiratory states were detected between the two experimental groups for different conditions stimulating mitochondrial respiration in the presence of malate and pyruvate without adenylates in intact (PN) or permeabilized cells (PL,N), saturating concentration of ADP in the presence of malate and pyruvate (PP) and succinate (SP) to supply electron to complex I and II, respectively. The uncoupled mitochondrial respiration (ET) rate increased in both the experimental groups indicating that the phosphorylation system is limiting OxPhos. By inhibiting the C-I with rotenone the uncoupled respiration rate decreased (R) in both the experimental groups. The contribution of the C-I to the ET respiration rate (ET-R) was determined by calculating the difference between ET and R respiration rates and was unchanged in both HepG2 cell suspensions. Furthermore, the respiration rate with C-IV substrates in the SCR group was similar to what observed in the *LGALS3 KO* HepG2 cells.

### *LGALS3* silencing reduces neutral lipid content in HepG2 cells

**Fig 3:**
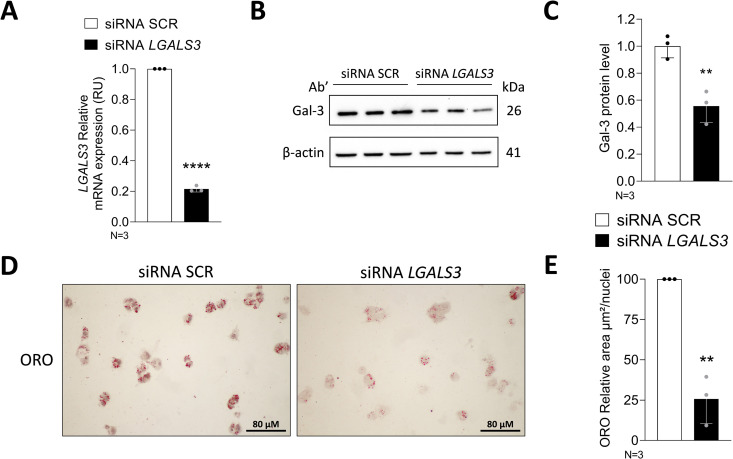
*LGALS3* silencing causes a significant reduction of the intracellular neutral lipid content. **A.** qRT-PCR analysis of *LGALS3* mRNA levels in HepG2 cells. Measurements were normalized to β-actin. Relative quantification was calculated by 2^‐ΔΔCt^ method. B. Western Blot analysis showing Gal-3 levels in siRNA SCR and siRNA *LGALS3* HepG2 cells. β-actin was used as loading control. C. Western blot quantification of Gal-3 related to the corresponding β-actin. D. Representative images (20x) from cells stained with ORO; nuclei were stained with Carazzi Hematoxylin. E. Quantification of intracellular ORO-stained area by ImageJ. Graphs represent N = 3 independent experiments. Unpaired t-test was performed to compare the two groups. ** p < 0.01, **** p < 0.0001. Bars represent mean ± SD. Abbreviations: SCR, scramble; ORO, Oil Red O.

In order to verify the impact of *LGALS3* silencing on neutral lipid content in HepG2 cells, Oil Red-O (ORO) staining was performed. In standard culture conditions, HepG2 cells display micro-vesicular cytoplasmic lipid accumulation. ORO staining confirmed the lipid accumulation in HepG2 cells in basal condition. Notably, following *LGALS3* knockdown, as confirmed by a significant down-regulation of mRNA and protein levels (Fig 3A–3C), HepG2 cells showed a strong reduction in neutral lipid content (Fig 3D–3E).

### *LGALS3* silencing reduces neutral lipid content in 3D spheroids

**Fig 4:**
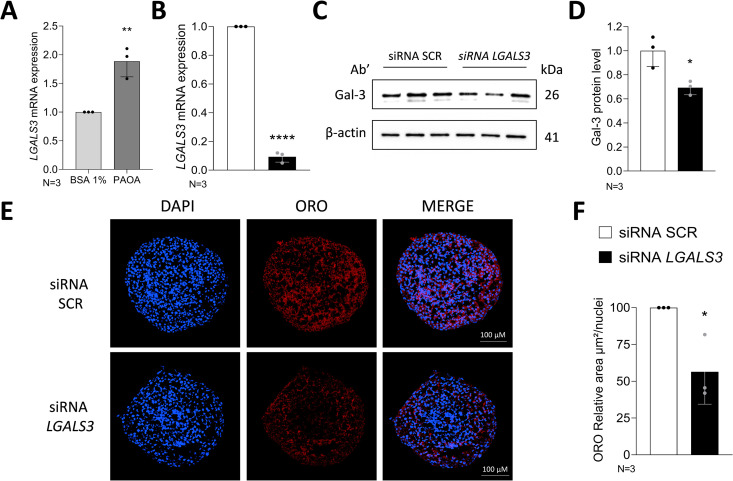
*LGALS3* silencing reduces the neutral fat accumulation in 3D spheroids. A. *LGALS3* mRNA levels in 3D spheroids composed of HepG2/LX-2 cells (ratio 24:1) treated with BSA 1% or a mix of palmitic acid and oleic acid 500 µM (1:2). β-actin was used as an endogenous control. Relative quantification was calculated by 2^‐ΔΔCt^ method. **B.** qRT-PCR analysis of *LGALS3* mRNA levels in siRNA SCR and siRNA *LGALS3* groups. Measurements were normalized to β-actin. Relative quantification was calculated by 2^‐ΔΔCt^ method. C. Western Blot analysis showing Gal-3 levels in siRNA SCR and siRNA *LGALS3*. β-actin was used as loading control. D. Western blot quantification of Gal-3 related to the corresponding β-actin. E. Representative images (20x) of spheroids sections stained with ORO; nuclei were stained with DAPI. E. Quantification of intracellular ORO-stained area by ImageJ. Graphs represent N = 3 independent experiments. Unpaired t-test was performed to compare the two groups. * p < 0.05, ** p < 0.01, **** p < 0.0001. Bars represent mean ± SD. Abbreviations: SCR, scramble; ORO, Oil red O; PAOA, palmitic acid + oleic acid (1:2).

Analysis of *LGALS3* expression patterns in human MASLD and liver cancer datasets (GSE135251, GSE126848, GSE124535, GSE130970 and The Cancer Genome Atlas Liver Hepatocellular Carcinoma) [38–41] revealed a significant up-regulation of *LGALS3* levels in MASH compared to healthy or MASLD tissues. Additionally, *LGALS3* expression was markedly elevated in HCC samples across multiple datasets (S1A–S1C Figs). To further elucidate whether Gal-3 might be involved in the regulation of fatty acid accumulation in MASLD, spheroids were incubated for 48 hours with a mix of palmitic and oleic acid (PAOA 1:2) to a final concentration of 500 µM. Having observed induction of steatosis and *LGALS3* mRNA levels up-regulation (Fig 4A) in 3D spheroids following exposure to a mix of palmitic acid and oleic acid, we next investigated whether *LGALS3* silencing could influence the neutral lipid content also in this 3D model of steatosis. Notably, qRT-PCR and Western blot analyses demonstrated a significant down-regulation of *LGALS3* mRNA and protein levels following *LGALS3* silencing in HepG2, LX-2 cells and 3D spheroids (Fig 3A–3C, S2 Fig, Fig 4B–4D). The intra-spheroid accumulation of fat was confirmed by ORO fluorescent staining. Consistently to what previously observed in 2D condition, the ORO staining showed a reduction of neutral lipid content in spheroids following *LGALS3* silencing, compared to the negative control scramble (SCR) (Fig 4E–4F).

### *LGALS3* silencing reduces type I collagen production

**Fig 5:**
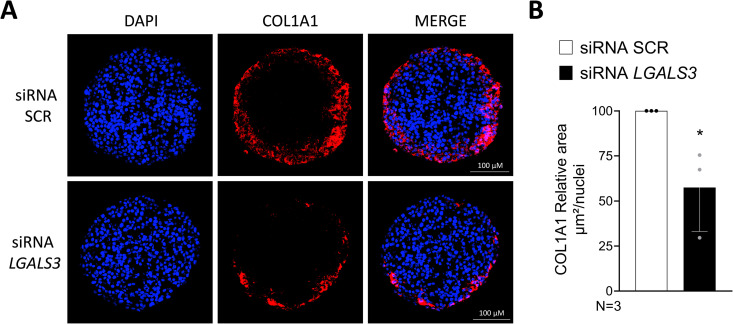
*LGALS3* silencing reduces collagen type I secretion. A. Immunofluorescence staining of COL1A1 (red), DAPI (blue), and merged images of 3D spheroids (HepG2/LX-2, 24:1) treated with a mix of palmitic acid and oleic acid 500 µM (PAOA 1:2). B. Quantification of COL1A1 levels by ImageJ normalized to number of nuclei. Unpaired t-test was performed to compare the two groups. * p < 0.05. Bars represent mean ± SD. Graphs represent N = 3 independent experiments. Abbreviations: SCR, scramble; COL1A1, type I collagen; PAOA, palmitic acid + oleic acid (1:2).

As previously reported, 3D spheroids treated for 48 hours with free fatty acids (PAOA) showed an increase in collagen type I alpha 1 (COL1A1) production [34]. Interestingly, the treatment with *LGALS3* siRNA significantly reduced the COL1A1 levels, compared to negative control SCR (Fig 5A–5B).

### Effect of *LGALS3* silencing on lipid metabolism

**Fig 6:**
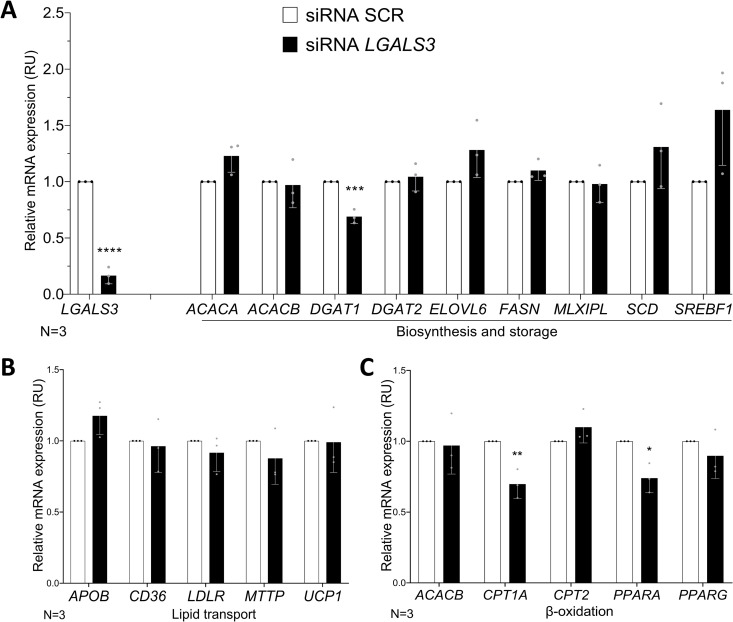
Gene expression analysis of genes involved in A) lipid biosynthesis and storage, B) transport and C) β-oxidation. Measurements were normalized to β-actin. Relative quantification was calculated by 2^‐ΔΔCt^ method. Groups were compared using unpaired two-tailed Student’s t-test and. Graphs represent N = 3 independent experiments. Values are shown as mean ± standard deviation. * p < 0.05, ** p < 0.01 *** p < 0.001, **** p < 0.0001.

To establish molecular mechanisms involved in the regulation of the effect of Gal-3 on lipid accumulation, we analysed the expression of genes encoding for enzymes crucial in the following pathways: a) lipid biosynthesis and storage: Acetyl-CoA Carboxylase Alpha (*ACACA*) and Beta (*ACACB*), Diacylglycerol O-Acyltransferase 1 (*DGAT1*) and 2 (*DGAT2*), Fatty Acid Elongase 6 (*ELOVL6*), Fatty Acid Synthase (*FAS*), MLX Interacting Protein Like (*MLXIPL*), Stearoyl-CoA Desaturase and Sterol Regulatory Element Binding Transcription Factor 1 (*SREBF1*); b) lipid transport: Apolipoprotein B (*APOB*), CD36 Molecule (*CD36*), Low Density Lipoprotein Receptor (*LDLR*), Microsomal Triglyceride Transfer Protein and Uncoupling Protein 1 (*MTTP*); c) β-oxidation: Carnitine Palmitoyltransferase 1A (*CPT1A*) and 2 (*CPT2*), Peroxisome Proliferator Activated Receptor Alpha (*PPARA*) and Gamma (*PPARG*). *LGALS3* silencing caused a significant reduction in the levels of *DGAT1*, *CPT1A* and *PPARA,* thus affecting lipid synthesis and fatty acid oxidation, respectively (Fig 6A–6C).

## Discussion

In the present study, we employed an *in vitro* model of MASLD, which consisted of 3D multilineage hepatic spheroids, composed of hepatic stellate LX-2 and hepatoma HepG2 cells, homozygous for the *PNPLA3* I148M sequence variant, the strongest genetic determinant of MASLD. Lipid accumulation and collagen secretion were induced in this model by incubation with a mixture of palmitic and oleic acid. The main findings stemming from the present study are: 1) Gal-3 inhibition is beneficial against steatosis and liver fibrosis in a 3D spheroid model; 2) genes involved in beta-oxidation may be affected without significant changes in fatty acid synthesis and lipid transport. Literature data regarding the effect of *LGALS3* ablation on hepatic steatosis, inflammation and liver fibrosis are still contradictory. Early studies by Nomoto *et al.* [28] demonstrated that *LGALS3*-deficient mice spontaneously developed steatosis and that the lack of Gal-3 induced greater hepatic lipid accumulation and injury in the model of choline deficient L-amino acid defined (CDAA) diet-induced NASH [29]. Moreover, Gal-3 deficiency, using the model of high fat diet-induced obesity (DIO) in mice, led to dysregulated glucose metabolism and inflammation [42]. On the contrary, another study [43] reported that *LGALS3* ablation protected from diet-induced MASH and attenuated inflammation, hepatocyte injury and fibrosis. Recently, Yu *et al.*[44] showed that, systemic inhibition of Galectin-3 by injection of TD139 improved hepatic steatosis and insulin resistance. With regard to liver fibrosis, ablation of Gal-3 in mice fed a high-fat diet led to marked liver steatosis, but attenuated liver inflammation and fibrosis [30]. In this regard, Henderson *et al.* [24] demonstrated that siRNA silencing of *LGALS3* expression in both mouse and human HSCs inhibited myofibroblast activation and procollagen (I) expression, markedly attenuating liver fibrosis. In fact, Belapectin, a Gal-3 inhibitor, has also been investigated in individuals with advanced fibrosis with promising results regarding lowering of hepatic venous pressure gradient and development of varices, but with no significant changes in liver fibrosis compared to placebo [27]. In this regard, an *in vitro* system, that can be used to examine the role of Gal-3 and its importance as a possible therapeutic target, is highly needed. Based on the contradictory *in vivo* findings, we sought to elucidate the molecular mechanisms of the role of *LGALS3* on 3D spheroid models. The study of MASLD cannot be limited to the analysis of the mechanisms involving hepatocytes without evaluating their interaction with non-parenchymal cells. Firstly, we generated the co-culture of hepatoma (HepG2) and hepatic stellate (LX-2) cells then, neutral fat accumulation and extracellular matrix proteins production were induced by free fatty acids exposure. After having successfully induced steatosis with a mixture of palmitic and oleic acid, we observed that *LGALS3* silencing resulted in lower hepatic neutral fat content. In individuals with CLD, Gal-3 levels are upregulated in advanced fibrosis (F3/F4) compared to lower levels of fibrosis, F0/F1 [45]. In our 3D *in vitro* model, higher type I collagen (COL1A1) content was observed after steatosis induction, recapitulating the human phenotype of liver steatosis leading to liver fibrosis. Interestingly, in line with the previously published data demonstrating that disruption of the *LGALS3* gene blocks myofibroblast activation and procollagen (I) expression *in vitro* and *in vivo* [24], the silencing of *LGALS3* in spheroids reduced COL1A1, as measured by immunofluorescence. Taken together, the results obtained in our experimental model confirm the previous studies suggesting that *LGALS3* ablation may prevent hepatic steatosis and attenuates liver fibrosis.

To further investigate the possible molecular mechanisms behind this phenotype, we analysed the mRNA levels of several players involved in lipid metabolism and β-oxidation. Our study demonstrated that the reduction in neutral lipid content and type I collagen production in 3D spheroids observed following *LGALS3* knockdown was accompanied a significant decrease in the mRNA levels of Diacylglycerol O-Acyltransferase 1 (*DGAT1*), involved in the fat storage through the esterification of exogenous fatty acids to glycerol [46], Carnitine Palmitoyltransferase 1A (*CPT1A*), an essential enzyme involved in the regulation of mitochondrial uptake of long-chain fatty acids for their subsequent β-oxidation and Peroxisome Proliferator Activated Receptor Alpha (*PPARA*), a ligand-activated transcription factor that regulates the peroxisomal β-oxidation pathway of fatty acids [47]. In line with the results demonstrating reduced neutral fat content following *LGALS3* ablation, lower *DGAT1* levels, the key enzyme in triglyceride synthesis, were observed. Accordingly, pharmacologic inhibition of *Dgat1*, a key enzyme of TG synthesis, with antisense oligonucleotides protected against hepatic steatosis in mice fed a high-fat diet [48]. While down-regulation of *DGAT1* in spheroids following *LGALS3* silencing could have protective effects in condition of hepatic steatosis, changes in *CPT1A* and *PPARA* mRNA levels, might be a consequence of the lipid content reduction or represent an independent effect exerted by Gal-3. Undoubtedly, the exact role of these enzymes in the regulation of lipid and collagen accumulation in *LGALS3* silenced cells requires further investigation. No changes were also observed in lipid uptake and transport, further pinpointing the specific effect of Gal-3 on fatty acid oxidation. In line with our observations, treatment with the specific Gal-3 inhibitor modified citrus pectin (MCP) was able to normalize CPT1A levels in HFD-fed rats [49]. Undoubtedly, this study comes with certain limitations. The exact mechanisms need to be revalidated in primary cells and more complex cell 3D models, involving more cell types such as Kupffer cells and endothelial cells, to elucidate the effect of Gal-3 in liver inflammation and fibrosis. Even though we identified a possible role of Gal-3 in the regulation of lipid metabolism, further studies are warranted to pin down the exact mechanisms behind the protective effect of Gal-3 downregulation.

**Fig 7:**
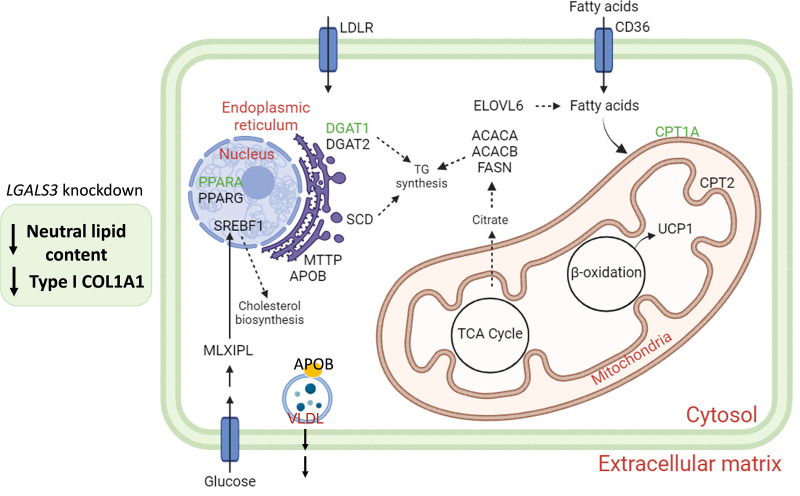
Summary representation of the effects of *LGALS3* silencing on the main enzymes involved in lipid biosynthesis, storage, transport and β-oxidation. The figure depicts the main genes involved in lipid biosynthesis, storage, transport and β-oxidation. Reduction in neutral lipid content and type I collagen production in 3D spheroids observed following *LGALS3* knockdown was accompanied by a significant decrease in the mRNA levels of *DGAT1, CPT1A* and *PPARA*, evidenced in green. Green: down-regulated genes. Abbreviations: KO, knockout; TG, triglycerides; TCA, tricarboxylic acid cycle. Created with BioRender.com.

In conclusion, the present study demonstrates that 3D spheroids represent a valid *in vitro* model to investigate the role of Gal-3 and its importance as a possible therapeutic target in MASLD. **Fig 7** summarizes the effects of *LGALS3* silencing on the main players involved in lipid metabolism.

## Supporting information

S1 Fig *LGALS3* expression trends in MASLD and HCC. **A.** Violin plots show *LGALS3* expression (log2(counts+1)) in GSE126848 and GSE135251 datasets, (***p < 0.001, borderline significance p ≈ 0.05). **B.** In GSE130970, *LGALS3* expression was elevated across fibrosis stages, with increases between stage 0 vs. stage 1 and stage 0 *vs.* stage 2 (**p < 0.01). **C.** *LGALS3* expression is markedly higher in HCC tissues compared to non-tumor controls in GSE124535 (***p < 0.001). In TCGA-LIHC, *LGALS3* expression shows an upward trend across fibrosis stages, with borderline significance between stage 1 and stage 4 (p ≈ 0.05). Red dots represent mean values; red error bars indicate the standard error of the mean (SEM). The Wilcoxon Rank-Sum Test was used to test *LGALS3* expression levels between different groups.
(TIF)

S2 Fig *LGALS3* mRNA and protein levels following *LGALS3* silencing in LX-2 cells. **A.** qRT-PCR analysis of *LGALS3* mRNA levels in LX-2 cells. Measurements were normalized to β-actin. Relative quantification was calculated by 2^‐ΔΔCt^ method. **B.** Western Blot analysis showing Gal-3 levels in siRNA SCR and siRNA *LGALS3*. β-actin was used as loading control. **C.** Western blot quantification of Gal-3 related to the corresponding β-actin.
(TIF)
