# Supplementary figures and images for: Galectin-3 inhibition ameliorates hepatic steatosis in a multilineage 3D spheroid model
Source: PLoS One. 2025 Jul 3;20(7):e0326373. doi: 10.1371/journal.pone.0326373 (PMC12225867; doi:10.1371/journal.pone.0326373)

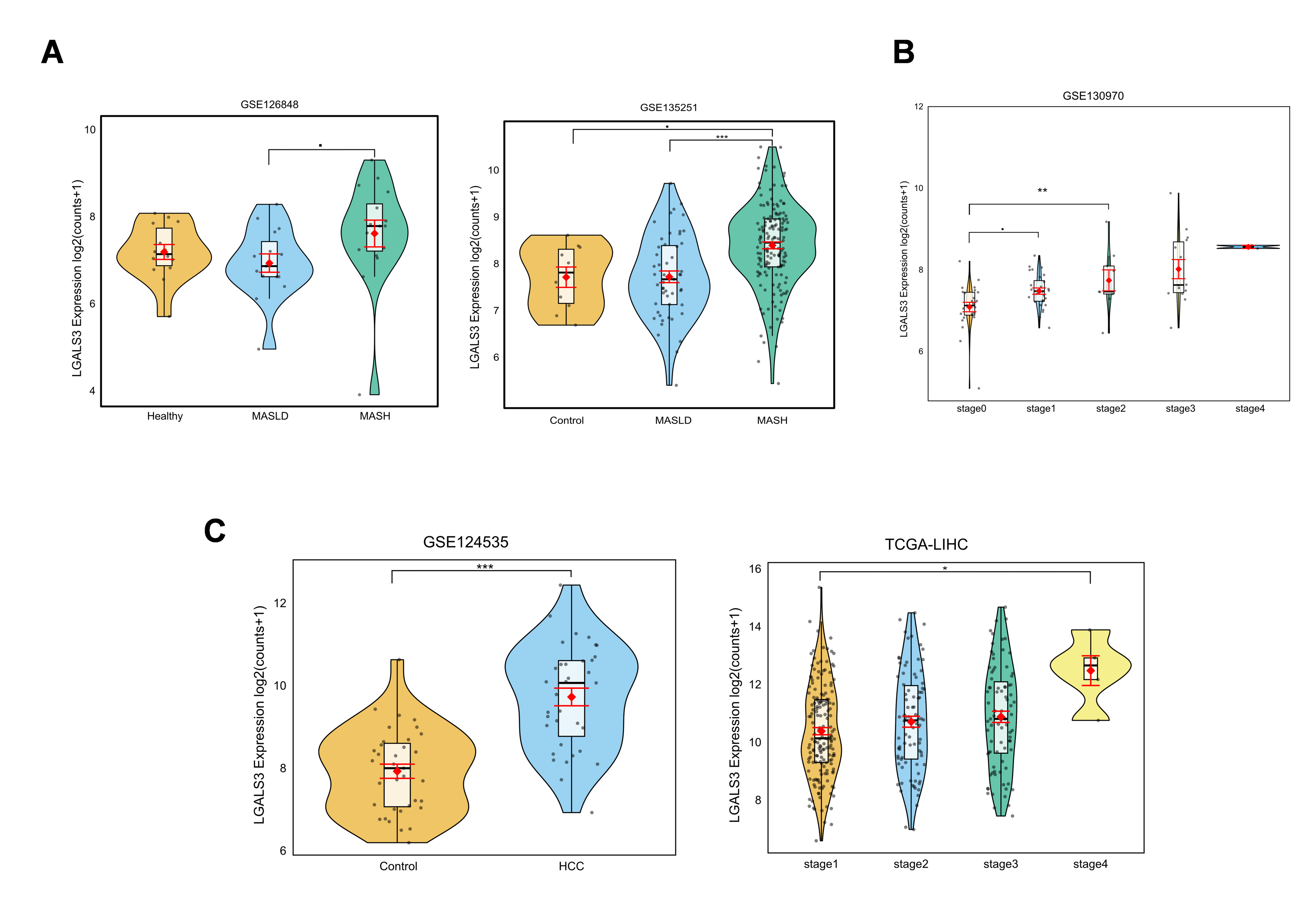

Supplement: S1 Fig — A. Violin plots show LGALS3 expression (log2(counts+1)) in GSE126848 and GSE135251 datasets, (***p < 0.001, borderline significance p ≈ 0.05). B. In GSE130970, LGALS3 expression was elevated across fibrosis stages, with increases between stage 0 vs. stage 1 and stage 0 vs. stage 2 (**p < 0.01). C. LGALS3 expression is markedly higher in HCC tissues compared to non-tumor controls in GSE124535 (***p < 0.001). In TCGA-LIHC, LGALS3 expression shows an upward trend across fibrosis stages, with borderline significance between stage 1 and stage 4 (p ≈ 0.05). Red dots represent mean values; red error bars indicate the standard error of the mean (SEM). The Wilcoxon Rank-Sum Test was used to test LGALS3 expression levels between different groups. (TIF) [file pone.0326373.s001.tif]

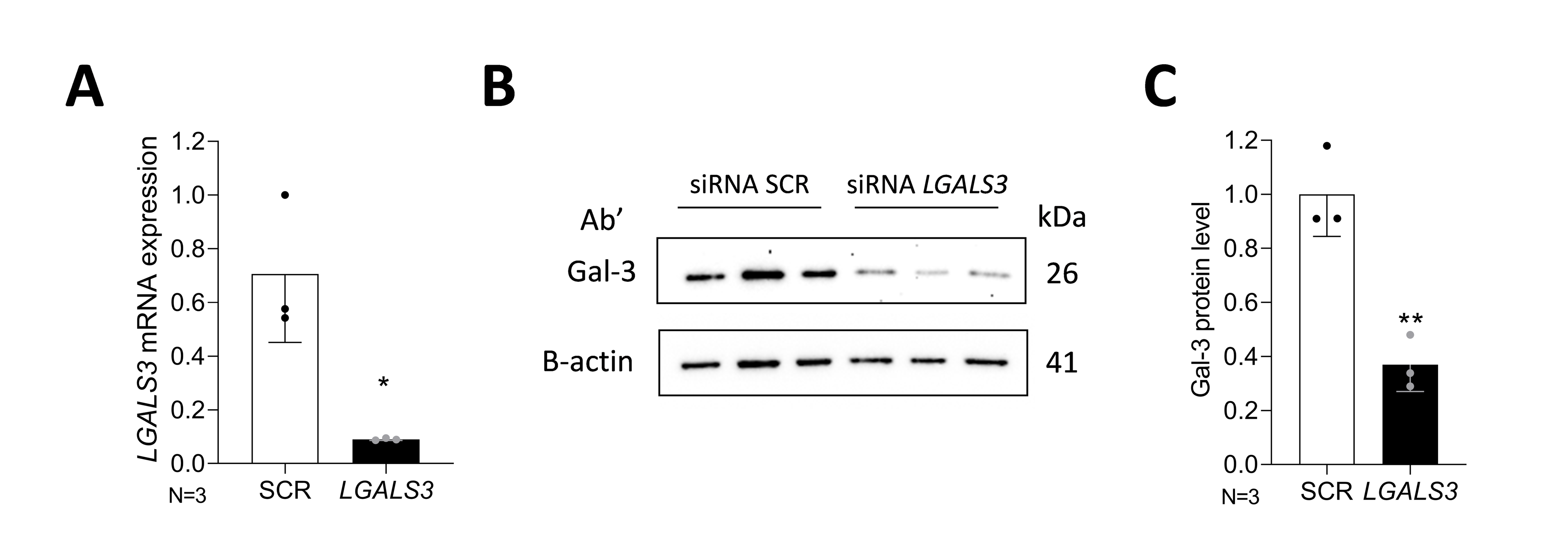

Supplement: S2 Fig — A. qRT-PCR analysis of LGALS3 mRNA levels in LX-2 cells. Measurements were normalized to β-actin. Relative quantification was calculated by 2‐ΔΔCt method. B. Western Blot analysis showing Gal-3 levels in siRNA SCR and siRNA LGALS3. β-actin was used as loading control. C. Western blot quantification of Gal-3 related to the corresponding β-actin. (TIF) [file pone.0326373.s002.tif]
